# Microsporogenesis and Pollen Morphology of Triploid and Tetraploid Rubber Trees (*Hevea brasiliensis*)

**DOI:** 10.3390/plants15172647

**Published:** 2026-08-29

**Authors:** Qiongxia He, Yujia Sun, Xu Wang, Xin Dong, Hongli Yang, Xiao Huang, Weiguo Li, Peng Qu, Huasun Huang, Yuanyuan Zhang

**Affiliations:** 1 Rubber Research Institute, Chinese Academy of Tropical Agricultural Sciences, Haikou 571101, China; 2 College of Tropical Crops, Yunnan Agricultural University, Kunming 665099, China; 3 School of Tropical Agriculture and Forestry, Hainan University, Haikou 572025, China

**Keywords:** *Hevea brasiliensis*, microsporogenesis, pollen, triploid, tetraploid

## Abstract

Polyploidy plays a key role in plant evolution and breeding, but the reproductive biology of polyploid rubber trees (*Hevea brasiliensis*) remains poorly understood. Here, we investigated microsporogenesis and pollen morphology in triploid and tetraploid rubber trees using acetic acid–magenta staining. Both polyploids displayed largely regular meiotic progression but exhibited abundant tetrad-stage abnormalities, including monads, dyads, triads, and unequal polyads. Tetraploids additionally exhibited chromosome misalignment, lagging chromosomes, asynchronous segregation, and multipolar spindles, with abnormal tetrads accounting for 24.52%. A total of more than 26,000 pollen grains (15,009 triploid and 11,774 tetraploid) were analyzed. In the triploid, 95.21% were normal-sized (30.7–43.3 µm), 2.21% were large (putative 2*n*; mean 47.2 µm), and 2.59% were super-large (putative 4*n*; mean 64.2 µm). In the tetraploid, 95.23% were normal-sized (31.7–46.7 µm), 4.75% were small (mean 19.6 µm), and only 0.025% were large (mean 55.3 µm). Normal-sized pollen from both polyploids was comparable to haploid pollen from diploids. Most large pollen grains exhibited wrinkled or irregular morphology, suggesting non-viability. Thus, functional male gametes in rubber tree polyploids are mostly reduced, and unreduced pollen is rare or nonviable. This study provides the first detailed description of the morphological sequence of microsporogenesis in triploid rubber tree and a cytological basis for understanding the low male fertility potential in breeding programs.

## 1. Introduction

Polyploidy, or genome doubling, has occurred repeatedly throughout plant evolution and is hypothesized to be a source of evolutionary innovation and species diversification. Phylogenomic analyses have revealed that two ancient whole-genome duplications occurred in the common ancestors of extant seed plants and extant angiosperms, respectively, and that these events contributed to the diversification of regulatory genes important to seed and flower development [1]. It also has potential in plant breeding for producing seedless polyploid cultivars and enhancing resistance or tolerance to biotic and abiotic stresses [2,3,4]. It is estimated that 15% of angiosperm and 31% of fern speciation events are accompanied by ploidy increases [5]. The production of unreduced gametes, rather than somatic chromosome doubling in the zygote, is considered the primary mechanism of polyploid formation [6,7]. Polyploidy can also be induced in vivo or in vitro through physical or chemical methods [8,9].

In flowering plants, although high-ploidy species such as hexaploid wheat (*Triticum aestivum* L.) [10] and *Brassica* L. [11] exist, triploids and tetraploids are among the most common polyploid types. Triploids are considered a reproductive barrier but also a bridge in polyploid formation, as they predominantly produce aneuploids along with a small proportion of triploids and tetraploids [12,13]. In contrast, tetraploids serve as a source of diploid gametes, which can be used to generate triploids and second-generation tetraploids [14].

The rubber tree (*Hevea brasiliensis* (Willd.) Müll.-Arg.) belongs to the genus *Hevea* in the family Euphorbiaceae and is a deciduous perennial tree crop indigenous to the Amazon rainforest in South America. With a chromosome number of 2*n* = 2*x* = 36, it is considered a paleotetraploid that has undergone at least one whole-genome duplication followed by diploidization [15,16]. The rubber tree is the only commercially cultivated rubber-producing plant worldwide [17]. Natural rubber is obtained from latex that flows from laticifers in the bark. It possesses high-performance properties, such as elasticity, resilience, heat dispersion, and abrasion resistance, which cannot be easily mimicked by synthetic polymers. As an important industrial raw material, it is widely used in national defense, transportation, healthcare, and daily necessities, and plays a significant role in economic and social development [18,19].

Both natural and artificially induced polyploids of rubber tree, including triploids and tetraploids, have been frequently obtained [20,21,22,23,24], providing abundant materials for polyploid breeding. Moreover, polyploid varieties have been selected and planted on a large scale [25,26]. Nevertheless, these varieties were predominantly obtained through one-time induction rather than via sexual crosses, and their use as parents for generating subsequent polyploid generations is severely constrained by fertility issues. Moreover, research on the reproductive biology of rubber tree polyploids remains extremely scarce. For female flowers, direct observation of megasporogenesis is difficult due to technical limitations. For male flowers, although a few studies have observed microsporogenesis in tetraploid rubber trees [16,27,28,29], they lack detailed descriptions, and no studies on triploid microsporogenesis have been reported. These gaps also hinder the utilization of male gametes from rubber tree polyploids.

In this study, microsporogenesis and pollen morphology of triploid and tetraploid rubber trees were observed and compared with those of diploids reported in previous studies, to explore the effects of polyploidy on microsporogenesis and pollen morphology variation and provide a basis for the sexual utilization of triploid and tetraploid rubber tree germplasm.

## 2. Results

### 2.1. Chromosome Counting of Triploid and Tetraploids

**Figure 1:**
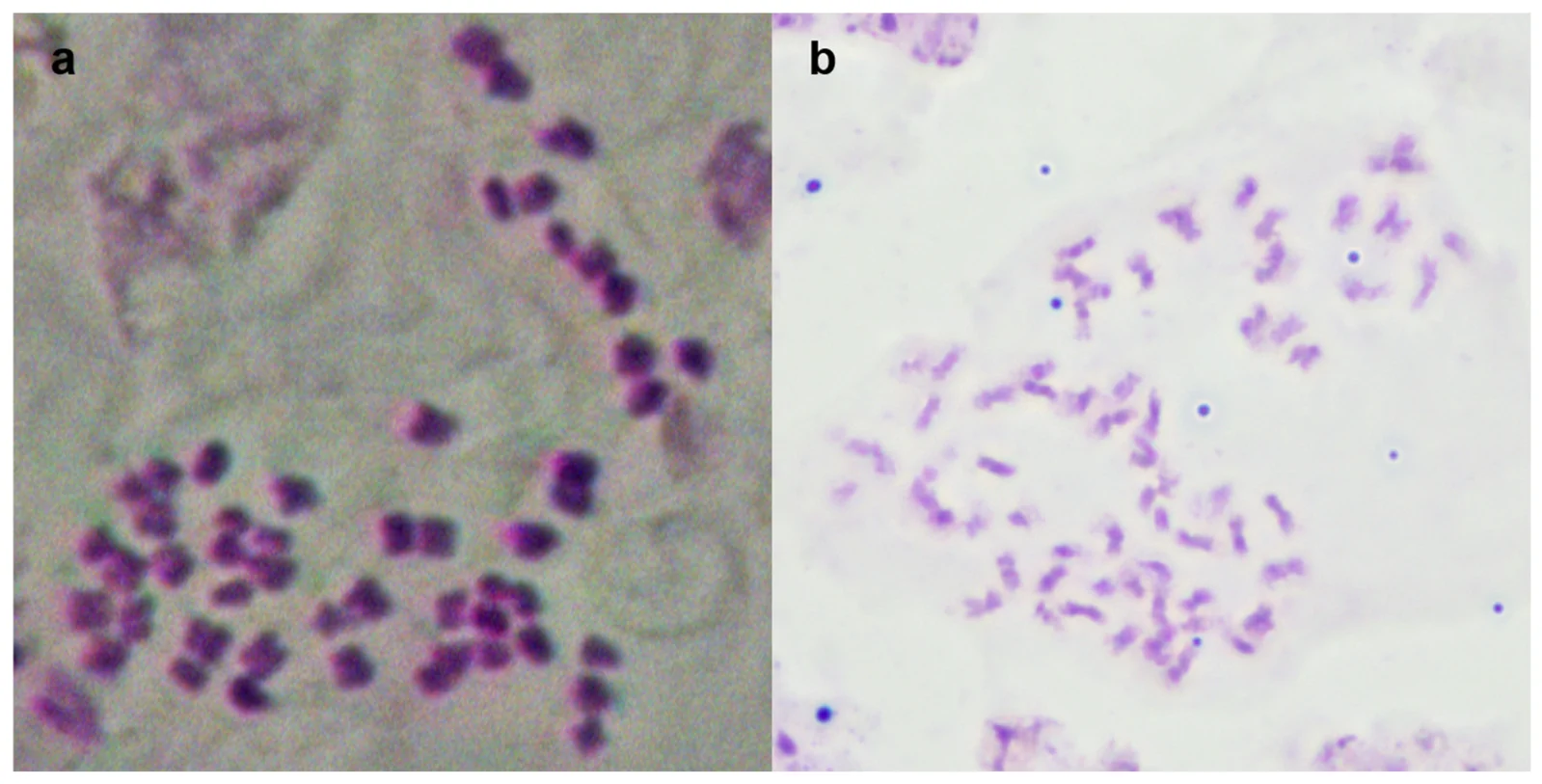
Chromosome counting of triploid and tetraploid. (**a**) Triploid (2*n* = 3*x* = 54); (**b**) Tetraploid (2*n* = 4*x* = 72).

To confirm the exact ploidy levels and exclude aneuploidy, we performed mitotic chromosome counting in leaf cells. The triploid plant consistently exhibited 54 chromosomes (2*n* = 3*x* = 54) in all metaphase cells examined (Figure 1a). The tetraploid plants showed 72 chromosomes (2*n* = 4*x* = 72) (Figure 1b), confirming that they are euploid tetraploids rather than aneuploids. No cells with chromosome numbers deviating from the expected multiples of 18 were observed in any of the samples. These results confirm that the triploid and tetraploid materials used in this study are genuine euploids, and that flow cytometry in previous study accurately reflected their ploidy levels.

### 2.2. Morphological Sequence of Microsporogenesis and Pollen Development

**Figure 2:**
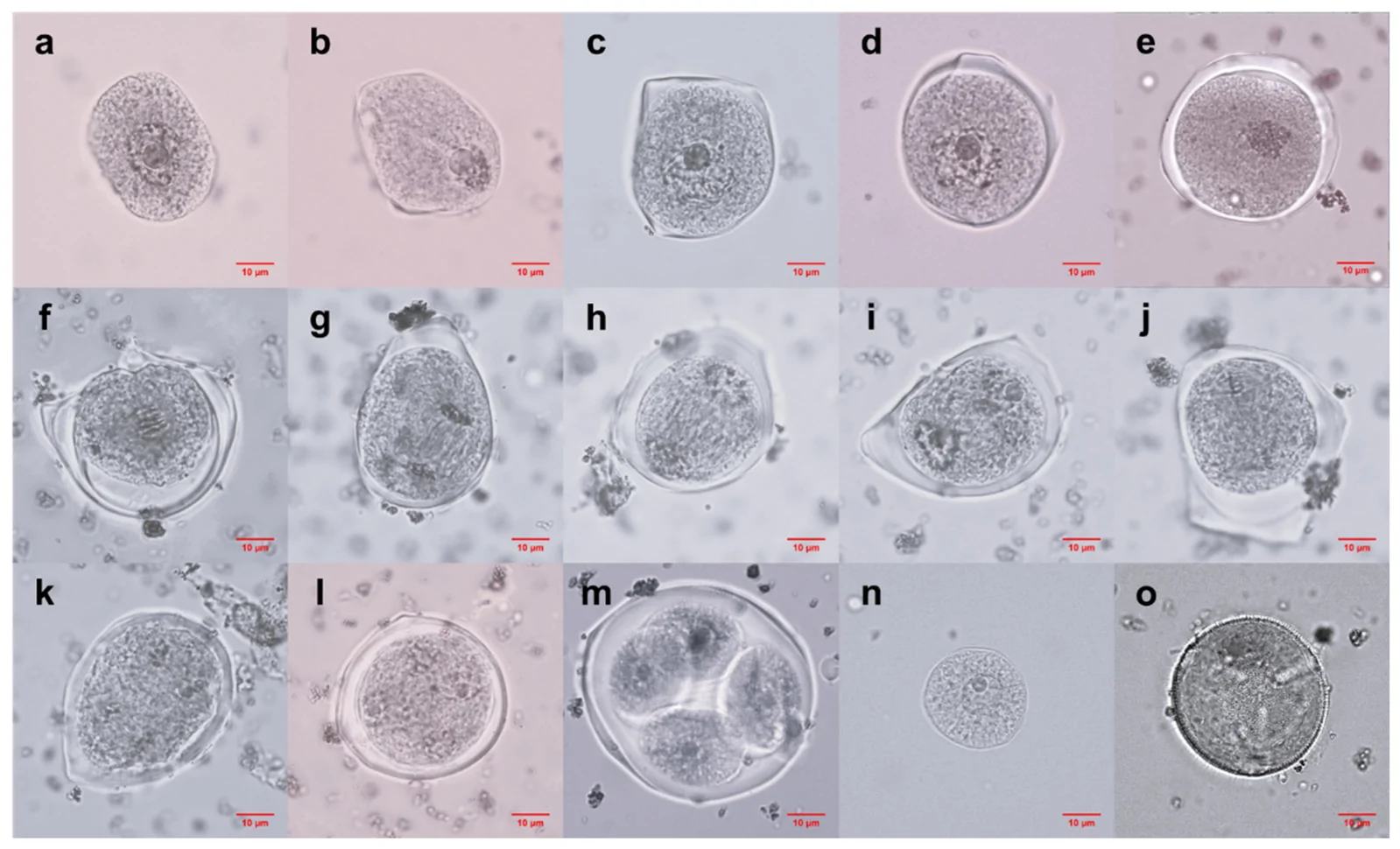
Meiotic stages observed during microsporogenesis in triploid rubber tree. (**a**) early leptotene; (**b**) late leptotene; (**c**) pachytene; (**d**) diplotene; (**e**) diakinesis; (**f**) metaphase I; (**g**) anaphase I; (**h**) telophase I; (**i**) prophase II; (**j**) metaphase II; (**k**) anaphase II; (**l**) telophase II; (**m**) tetrad; (**n**) microspore; (**o**) pollen grain.

**Figure 3:**
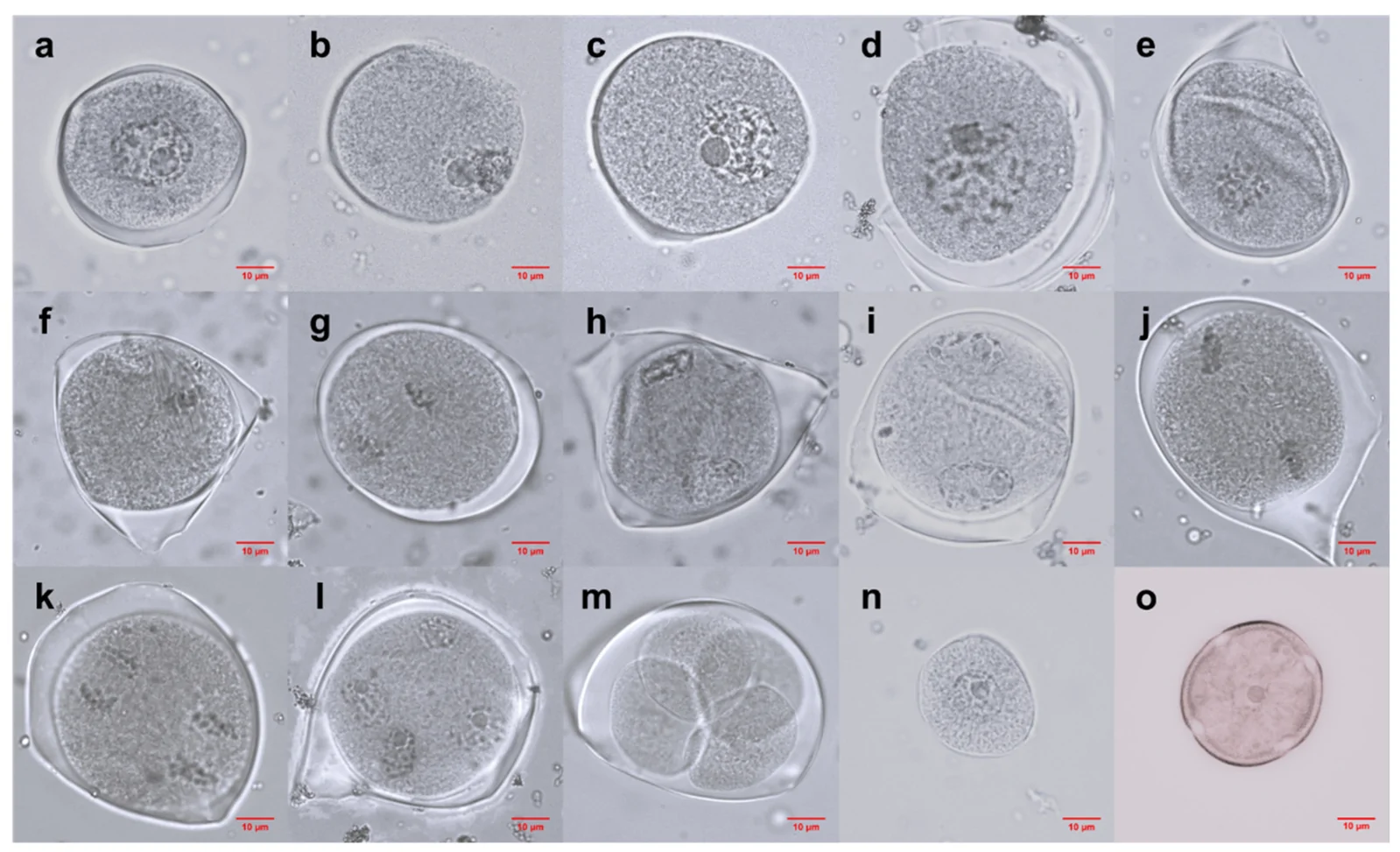
Meiotic stages observed during microsporogenesis in tetraploid rubber trees. (**a**) early leptotene; (**b**) late leptotene; (**c**) pachytene; (**d**) diplotene; (**e**) diakinesis; (**f**) metaphase I; (**g**) anaphase I; (**h**) telophase I; (**i**) prophase II; (**j**) metaphase II; (**k**) anaphase II; (**l**) telophase II; (**m**) tetrad; (**n**) microspore; (**o**) pollen grain.

In both triploids and tetraploids, the majority of pollen mother cells exhibited the complete morphological sequence of meiotic stages from leptotene to tetrad formation, and the progression of these stages was synchronous, just as in diploids [30,31]. Representative images of the main meiotic stages in triploids and tetraploids are shown in Figure 2 and Figure 3. Prophase I was divided into five stages. In early leptotene, the nucleolus was distinct and surrounded by darkly stained chromatin (Figure 2a and Figure 3a). In late leptotene, the “bouquet” structure of chromatin was evident (Figure 2b and Figure 3b). During pachytene, the chromatin became shorter, thicker and more visible (Figure 2c and Figure 3c). During diplotene, chromatin further condensed and shortened, homologous chromosomes appeared to form a semi-circular structure and were dispersed around the nucleolus (Figure 2d and Figure 3d). In diakinesis, the nucleolus disappeared and the chromosomes condensed into short rod-shaped dots that were evenly dispersed (Figure 2e and Figure 3e). In metaphase I, chromosomes were neatly aligned on the equatorial plate, and spindle fibers emitted from the two poles were clearly visible (Figure 2f and Figure 3f). In anaphase I, homologous chromosomes separated and moved toward the poles (Figure 2g and Figure 3g). In telophase I, homologous chromosomes reached the poles and small nucleoli appeared, while the spindle fibers were disappearing (Figure 2h and Figure 3h). In prophase II, the small nucleoli at both poles aggregated into large nucleoli and fibers disappeared (Figure 2i and Figure 3i). In metaphase II, chromosomes at both ends of the cell were arranged on the equatorial plate (Figure 2j and Figure 3j). In anaphase II, sister chromatids separated and moved to the four poles almost synchronously (Figure 2k and Figure 3k). In telophase II, nucleoli reappeared at the four poles (Figure 2l and Figure 3l). The above description documents the morphological progression through meiotic stages as observed in the majority of pollen mother cells. We do not claim that chromosome pairing behavior in these polyploids is identical to that of diploids. Chromosome configuration analysis at the level of bivalents, trivalents, or quadrivalents could not be performed due to the extreme condensation and dense packing of chromosomes at these stages in our fixed samples.

During the tetrad stage, chromosomes disappeared and a callose wall formed, enclosing the four poles (Figure 2m and Figure 3m). Subsequently, the outer callose wall of the microspore mother cell dissolved, releasing microspores that were significantly smaller than the mother cell (Figure 2n and Figure 3n). After microspores developed into pollen grains, granular particles were visible on the pollen wall, and the pollen grains were considerably larger than the microspores. Pollen grains from triploids had three germ pores (Figure 2o), whereas most tetraploid pollen grains had four germ pores (Figure 3o).

### 2.3. Abnormal Microsporogenesis and Pollen Development

**Figure 4:**
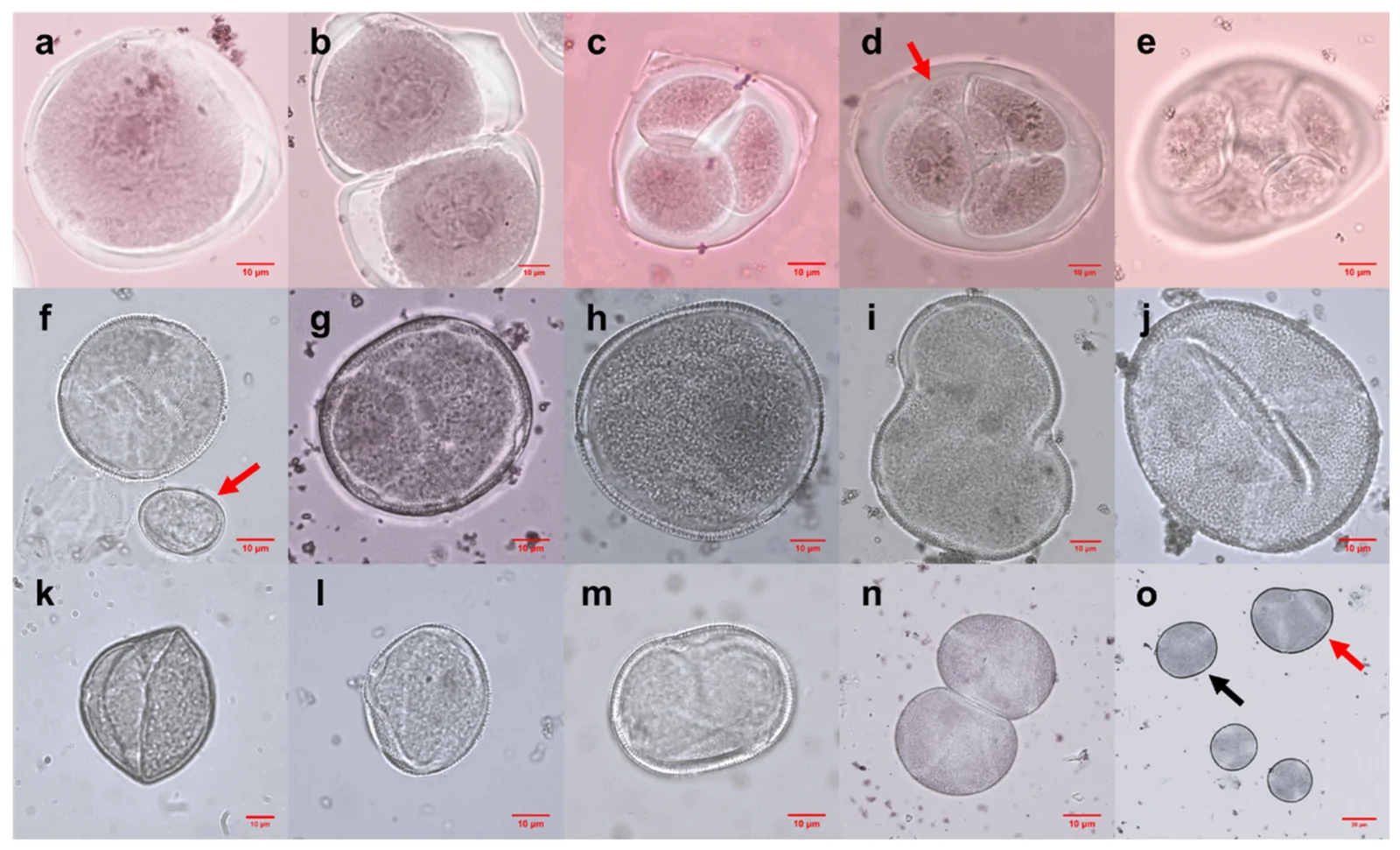
Abnormal phenomena during microsporogenesis and abnormal pollens of triploid *Hevea brasiliensis*. (**a**) Monad; (**b**) Dyad; (**c**) Triad; (**d**) Uneven pentad (microcyte indicated by the red arrow); (**e**) Hexad; (**f**) Normal pollen and small pollen (indicated by the red arrow); (**g**) Large pollen; (**h**) Super-large pollen; (**i**) Irregularly shaped super-large pollen; (**j**) Wrinkled super-large pollen; (**k**) Shriveled pollen; (**l**) Unsaturated pollen; (**m**) Conjoined pollen; (**n**) Adhesive pollen; (**o**) Normal pollen, large pollen (indicated by the black arrow), and super-large pollen (indicated by the red arrow).

In the triploid, abnormalities during microsporogenesis were mainly observed at the tetrad stage, including monads, dyads, triads, and unequal pentads and hexads (Figure 4a–e). Owing to the limited sample size, the proportion of abnormal tetrads was not calculated. During the pollen stage, in addition to normal pollen, abnormal sizes and shapes were observed, including small, large, super-large, irregularly shaped, wrinkled, shriveled, unsaturated, conjoined, and adhesive pollen grains (Figure 4f–n). Normal, large, and super-large pollen grains were also observed simultaneously in the same field of view (Figure 4o). It should be noted that the limited availability of triploid pollen mother cells at early meiotic stages precluded systematic observation of chromosome behavior during metaphase I and anaphase I. However, the high frequency and diversity of tetrad abnormalities observed in the triploid strongly suggest that such irregularities originated from defective chromosome segregation and cytokinesis during the first and second meiotic divisions.

**Figure 5:**
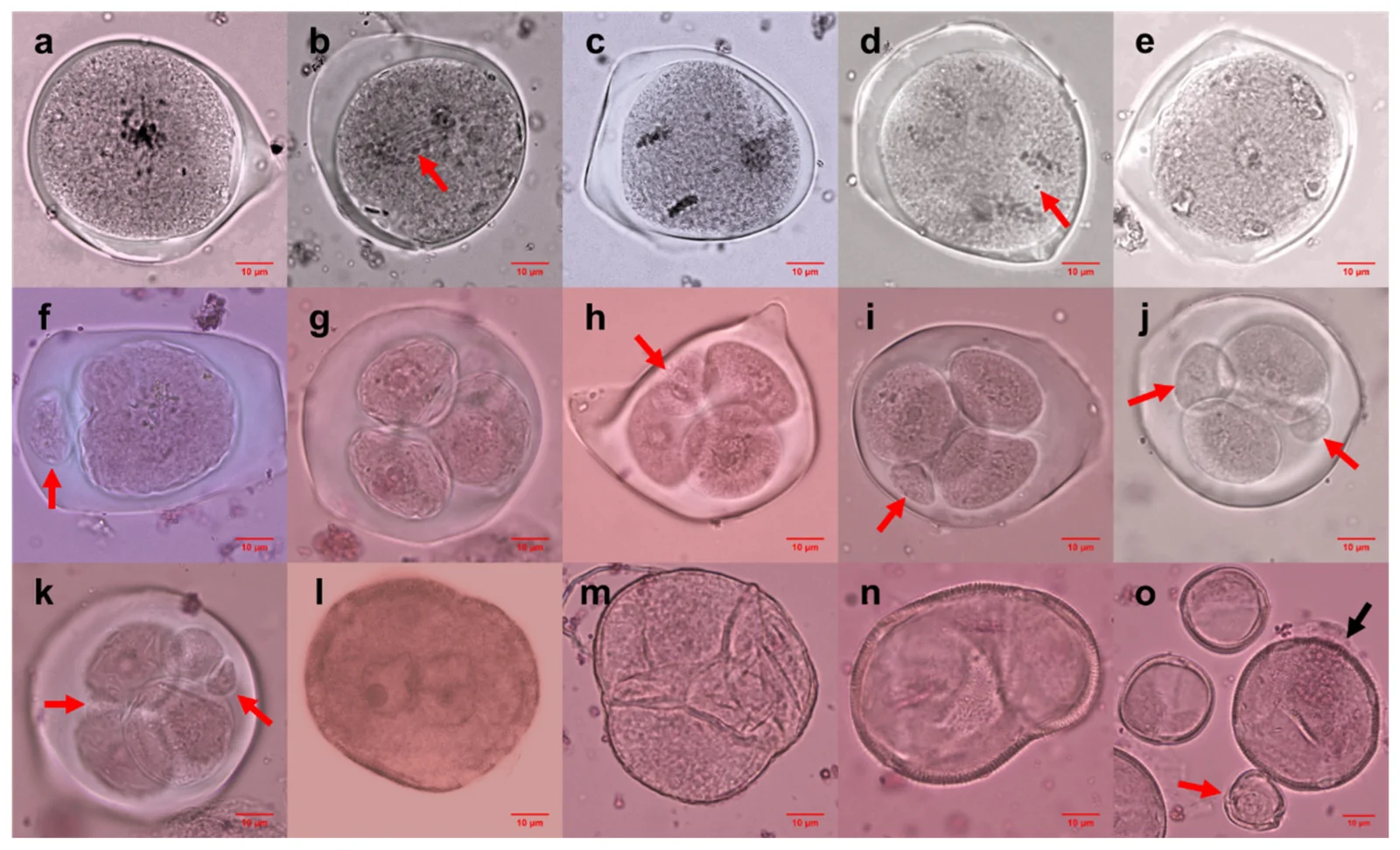
Abnormal phenomena during microsporogenesis and abnormal pollens of tetraploid *Hevea brasiliensis*. (**a**) Chromosome misalignment at metaphase I; (**b**) Lagging chromosome at anaphase I (indicated by the red arrow); (**c**) Asynchronous chromosome separation at anaphase II; (**d**) Lagging chromosome at anaphase II (indicated by the red arrow); (**e**) Multipolar poles at telophase II; (**f**) Uneven dyad (microcyte indicated by the red arrow); (**g**) Triad; (**h**) Uneven tetrad (microcyte indicated by the red arrow); (**i**) Uneven pentad (microcyte indicated by the red arrow); (**j**) Uneven hexad (microcyte indicated by the red arrow); (**k**) Uneven heptad (microcyte indicated by the red arrow); (**l**) Large pollen; (**m**) Wrinkled large pollen; (**n**) Irregularly shaped large pollen; (**o**) Normal pollens, large pollen (indicated by the black arrow) and small pollen (indicated by the red arrow).

In the tetraploid, numerous abnormalities were observed during microsporogenesis, including chromosome misalignment at metaphase I, lagging chromosome at anaphase I, asynchronous chromosome separation at anaphase II, lagging chromosomes at anaphase II, and multipolar poles at telophase II (Figure 5a–e). At the tetrad stage, abnormalities similar to those in the triploid were observed, including unequal dyads, triads, tetrads, pentads, hexads, and heptads (Figure 5f–k). These early meiotic irregularities are considered to be the direct causes of the subsequent tetrad abnormalities, as they lead to unequal chromosome distribution and defective cytokinesis during the first and second meiotic divisions. A total of 2276 microspore mother cells were observed at the tetrad stage, of which 558 were abnormal tetrads, accounting for 24.52%. At the pollen stage, large pollen grains, including wrinkled and irregularly shaped ones, were observed, but no super-large pollen was found, unlike in the triploid (Figure 5l–n). Normal, small, and large pollen grains were simultaneously present in the same field of view (Figure 5o).

Based on their occurrence stages, the abnormalities observed in this study (with direct evidence from tetraploids and indirect evidence from triploids) were classified into three categories. (1) Meiotic chromosome behavior abnormalities: including chromosome misalignment at metaphase I, lagging chromosomes at anaphase I and II, asynchronous chromosome separation at anaphase II, and multipolar poles at telophase II (Figure 5a–e). (2) Tetrad formation abnormalities: including monads, dyads, triads, unequal tetrads, pentads, hexads, and heptads (Figure 4a–e and Figure 5f–k), which arose from defective cytokinesis or unequal chromosome distribution during meiosis I and II. (3) Pollen morphology abnormalities: including small, large, super-large, irregularly shaped, wrinkled, shriveled, unsaturated, conjoined, and adhesive pollen grains (Figure 4f–n and Figure 5l–n). Quantitative analysis of chromosome configurations at diakinesis and metaphase I could not be performed due to technical limitations as detailed in Section 4.2, which prevented unambiguous identification of individual chromosome associations.

### 2.4. Abnormal Pollen Statistics and Pollen Grain Diameter Analysis

Pollen grains were classified into size classes based on visual inspection under the microscope, with normal-sized pollen in each field of view serving as the internal reference. This visual classification was subsequently verified by diameter measurements using ImageJ. The diameter ranges and mean values for each class are summarized in Table 1. Notably, the diameters of different size classes overlapped to some extent (e.g., normal pollen up to 43.30 μm and large pollen down to 39.20 μm in the triploid), confirming that visual morphology (relative size and shape) was the primary criterion for classification, with measurement serving as a complementary validation tool.

**Figure 6:**
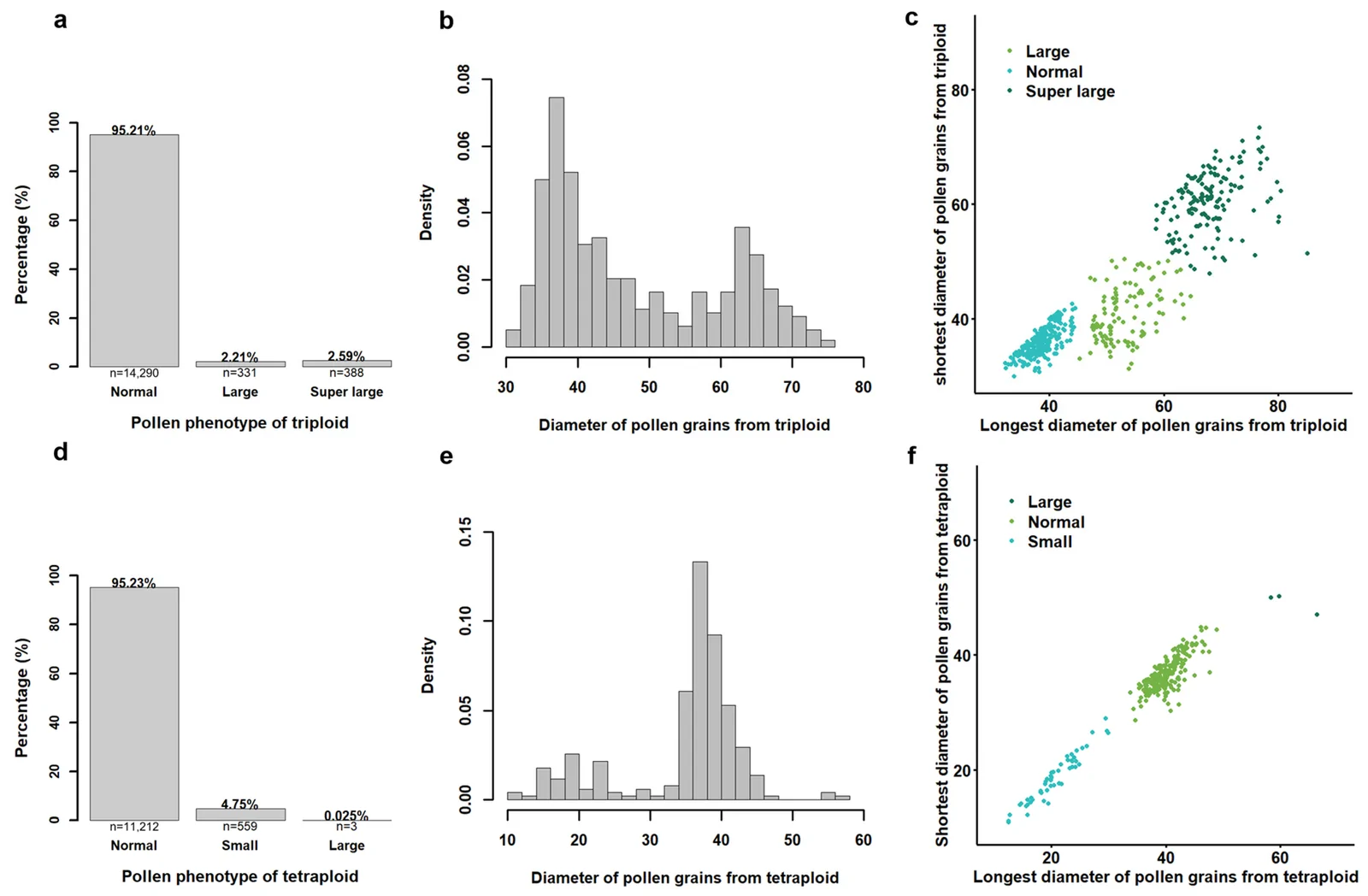
Analysis of pollen grain size classes and diameter measurements. (**a**) Proportion of pollen grains of different sizes from triploid; (**b**) Histogram of average pollen grain diameter from triploid; (**c**) Scatter plot of pollen grain diameters from triploid; (**d**) Proportion of pollen grains of different sizes from tetraploid; (**e**) Histogram of average pollen grain diameter from tetraploid; (**f**) Scatter plot of pollen grain diameters from tetraploid.

For the triploid, a total of 15,009 pollen grains were counted, of which 95.21% were normal-sized, with diameters ranging from 30.71 to 43.30 μm and a mean of 37.19 μm (Table 1). Large pollen accounted for 2.21%, with diameters ranging from 39.20 to 55.71 μm and a mean of 47.17 μm. Super-large pollen accounted for 2.59%, with diameters ranging from 56.55 to 75.06 μm and a mean of 64.16 μm. Small pollen grains were extremely rare, and their proportion and diameter have not been calculated (Figure 6a). Both the histogram of mean diameter (Figure 6b) and the scatter plot of pollen diameter based on maximum and minimum diameters (Figure 6c) show that pollen grains could be divided into three groups.

For the tetraploid, a total of 11,774 pollen grains were counted, of which 95.23% were normal-sized, with diameters ranging from 31.66 to 46.65 μm, and a mean of 38.39 μm (Table 1). Small pollen accounted for 4.75%, with diameters ranging from 11.76 to 29.20 μm, and a mean of 19.59 μm. Large pollen accounted for only 0.025%, with diameters ranging from 54.17 to 56.71 μm and a mean of 55.30 μm (Figure 6d). As in the triploid, both the histogram (Figure 6e) and the scatter plot (Figure 6f) indicated three distinct size groups.

### 2.5. Preliminary Determination of Pollen Viability

**Figure 7:**
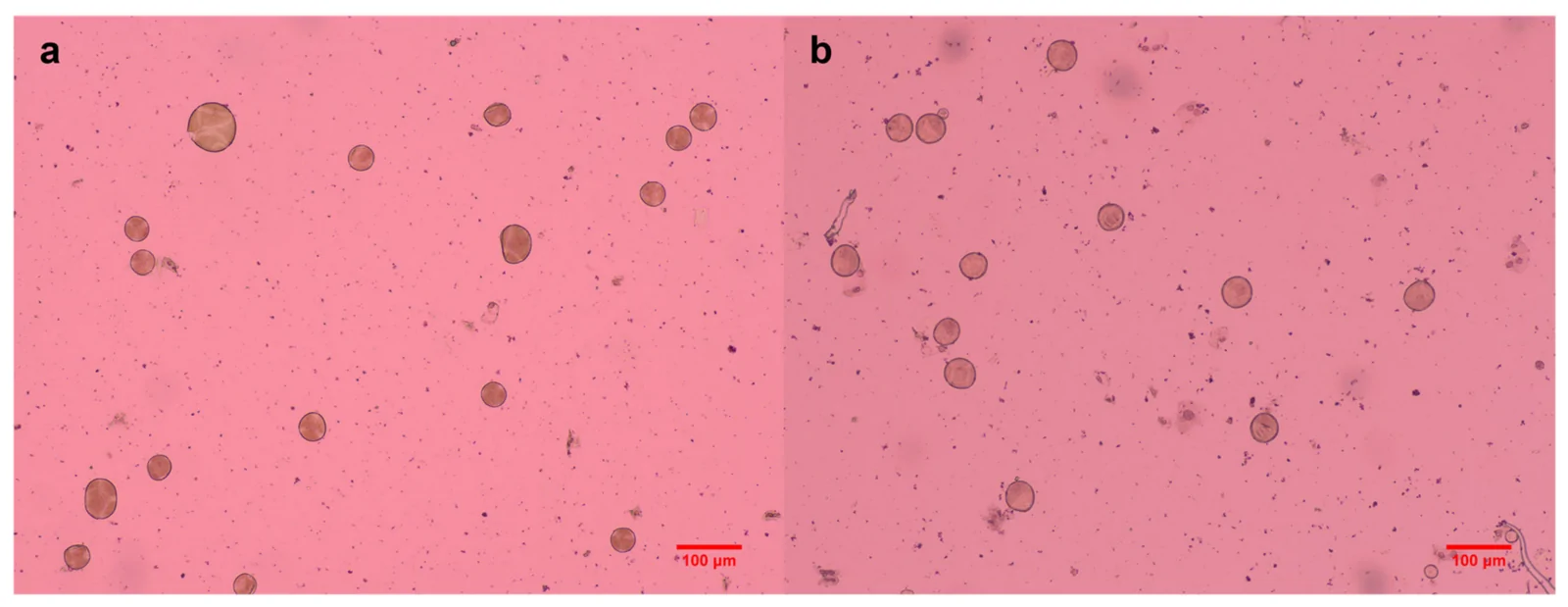
Detection of pollen viability by acetic acid magenta staining. (**a**) Pollen grains from triploid; (**b**) Pollen grains from tetraploid.

Based on acetic acid–magenta staining, we further evaluated pollen staining status as an indicator of cytoplasmic integrity. In this section, large and super-large pollen grains are interpreted as putative 2*n* and 4*n* pollen, respectively, based on the diameter relationships established in diploid rubber trees (see Section 2.4 and Section 3). Pollen grains exhibiting normal morphology, regardless of size class (normal, large, or super-large), were uniformly stained with acetic acid–magenta (Figure 7). In contrast, small, shriveled, and malformed pollen grains remained unstained. This observation suggests that the putative unreduced (2*n* and 4*n*) pollen grains possess intact plasma membranes, a prerequisite for viability, although staining-based methods may overestimate actual germination capacity (see Section 3 for further interpretation).

## 3. Discussion

Ploidy changes are often accompanied by fertility alterations, particularly in odd-ploidy plants. Most triploids tend to be sterile [32]. However, some fertile triploids undergo extensive chromosomal irregularities during meiosis and produce normal, unreduced, and aneuploid gametes, thereby playing a crucial role in enhancing plant diversity. In Chinese chives, irregular chromosome arrangement at metaphase I, lagging chromosomes and bridges at anaphase I, uneven chromosome distribution, and asynchronous cytokinesis have been observed. After meiosis, microspores with different nuclei per tetrad, small nucleated microspores in polyads, enucleate microspores, and tetrads containing one small and three large microspores were documented. In addition to normal pollen grains, miniature enucleate pollen, large pollen grains with two nuclei, normal-sized pollen with zero, one, or two nuclei, and large enucleate pollen grains were observed [33]. In *Populus*, lagging chromosomes and chromosome bridges at anaphase I, micronuclei at telophase I, parallel and fused spindles at metaphase II, lagging chromosomes at anaphase II, and premature cytokinesis during the first and second meiotic division occurred; dyads, triads, tetrads and polyads were observed in the triploid, and the diameter range of pollen grains was much larger than that of diploids. Additionally, trisomic individuals were detected in the 2*x* × 3*x* hybrid progeny [34]. In triploid *Cymbidium*, meiotic abnormalities such as asynchrony, lagging chromosomes, chromosome bridges, and abnormal spindle orientation occurred during microsporogenesis, resulting in the dyads, triads, equal- and unequal-sized tetrads, and ultimately producing 1*x*, 1*x*–2*x*, 2*x*, 2*x*–3*x*, and 3*x* male gametes. After being crossed with diploids, progeny with a wide range of ploidy levels were obtained, including diploids, 2*x*–3*x* aneuploids, triploids, and tetraploids, indicating that the male gametes from the sexual triploid were fertile and could fertilize egg cells [12]. Baldwin [27] reported that the triploid clone 5535 of *H. guianensis* (another *Hevea* species) exhibited irregular meiosis and suggested that the plant might be hexaploid rather than triploid. In detail, bivalents and univalents were typically present, although occasionally 27 bivalents were observed at diakinesis. Metaphase I was consistently erratic, with frequent precocious anaphase movement. At metaphase II, groups of several chromosomes were common, and up to 34 entities were found. Tetrads of four apparently normal microspores were rarely produced; instead, a single pollen mother cell typically gave rise to two giant and two smaller microspores of varying sizes, along with one-, two-, three- and four-minute microspores. As many as five nucleoli of diverse sizes were often observed. Consistent with these studies, we also found numerous abnormalities during microsporogenesis in the triploid rubber tree, such as monads, dyads, triads, unequal pentads, and hexads, as well as pollen grains of abnormal sizes and shapes, such as small, large, super-large, irregularly shaped, wrinkled, shriveled, unsaturated, conjoined, and adhesive pollen. Due to the limited number of well-spread pollen mother cells at early meiotic stages in our triploid samples, we were unable to directly observe chromosome behavior at metaphase I and anaphase I. Nevertheless, the variety and frequency of tetrad abnormalities observed provide compelling indirect evidence that irregular chromosome segregation and defective cytokinesis occurred during meiosis I and II. This inference is further supported by the direct observation of chromosome misalignment, lagging chromosomes, and multipolar spindles in tetraploid samples (Figure 5a–e), where the same categories of tetrad abnormalities were observed (Figure 5f–k). Together, these lines of evidence indicate that the tetrad-stage abnormalities in both polyploids are the consequence of irregularities during earlier meiotic divisions. It is important to emphasize that our description of microsporogenesis is morphological in nature. While the majority of pollen mother cells in both triploids and tetraploids progressed through the same sequence of morphological stages as observed in diploids, this observation does not imply that chromosome pairing configurations (e.g., bivalents, trivalents, quadrivalents) are identical to those of diploids. Our data do not allow conclusions about the underlying pairing behavior. This study provides the first systematic observation and description of microsporogenesis, pollen development, and pollen morphology in triploid rubber tree.

Autotetraploid usually exhibits weaker vitality than diploid, such as dwarfism and reduced yield [35,36,37,38], and has little direct utility. Therefore, tetraploids are often used to hybridize with diploids to produce triploids. For example, allotriploid loquat can be obtained via hybridization between tetraploids (female parent) and diploid wild species, with some aneuploids as secondary products [39]. Using the same strategy, three triploids were obtained from the hybridization between the autotetraploid variety ‘Chenguang’ and the diploid variety ‘Dongzao’ of Chinese jujube, where ‘Chenguang’ served as the male parent and one diploid was also produced [40]. This breeding strategy has also been attempted in rubber trees. Lu et al. [41] performed diallel hybridization between tetraploid and diploid plants and observed the flowering and reproductive organ characteristics of tetraploid, obtained fruits and seeds; however, the ploidy of the offspring was not identified. Although other studies have aimed to obtain triploids through polyploid hybridization, they have only reported microsporogenesis and pollen morphology in tetraploids, where abnormal phenomena were documented. For instance, in the tetraploid clone RRII105 [29], at metaphase I, besides predominant bivalent formation, univalents, trivalents and quadrivalents were observed; meanwhile, anaphase I showed unequal segregation, laggards, micronuclei, etc., whereas the subsequent division showed no abnormalities. Also, pollen grains of tetraploid RRII105 were bigger, and 30% of them had four germ pores. On the other hand, tetraploid plant of clone 3065 [28] showed a certain percentage of normal first meiotic divisions without laggards at anaphase I, but presented abnormal tetrads containing four microspores and one to four microcytes, and the pollen grains had four germ pores and arrested at the one-nucleus stage. In the present study, chromosome misalignment at metaphase I, lagging chromosomes at anaphase I, asynchronous chromosome separation at anaphase II, lagging chromosomes at anaphase II, and multipolar spindles at telophase II were observed during microsporogenesis, some of which resemble findings in the tetraploid RRII105. Meanwhile, unequal dyads, triads, unequal tetrads, pentads, hexads, and heptads were found at the tetrad stage, as in the tetraploid clone 3065. Furthermore, for the first time, large, wrinkled large, irregularly shaped large, small pollen were observed and described in tetraploid rubber tree.

Pollen grain size can serve as an indirect indicator of ploidy level. Generally, the diameter of unreduced (2*n*) pollen grains is about 1.2–1.4 times that of reduced (*n*) pollen grains in diploid plants [42,43,44,45,46,47]. This relationship also applies to rubber trees. Zhang et al. [31] found that the mean diameters of large pollen grains (53.23 μm) and super-large pollen grains (66.59 μm) produced by a diploid clone were 1.35 and 1.69 times that of reduced pollen grains (39.38 μm), and they could be identified as 2*n* pollen and 4*n* pollen, respectively. In this study, the mean diameter of normal pollen grains produced by triploid and tetraploid rubber trees was 37.19 μm and 38.39 μm, respectively, almost identical to that of normal diploid pollen. Moreover, the mean diameters of large and super-large pollen grains from the triploid were 47.17 μm and 64.16 μm, respectively, similar to those of the diploid, and can be identified as 2*n* pollen and 4*n* pollen, indicating that triploids produce pollen types comparable to those of diploids with abnormal meiosis. In tetraploids, the mean diameters of small and large pollen grains were 19.59 μm and 55.30 μm, respectively; small pollen grains may be derived from microcytes, while large pollen grains may originate from dyads and triads. The observed tetrad abnormalities—monads, dyads, triads, tetrads, pentads, and hexads—provide a mechanistic basis for the production of pollen grains with different chromosome numbers. In triploid rubber tree (2*n* = 3*x* = 54, where *x* = 18 based on the diploid chromosome number 2*n* = 2*x* = 36), hexad-type division yields gametes with *x* = 18 chromosomes (*n* = *x*); tetrad-type division yields gametes with 1.5*x* = 27 chromosomes; triad formation yields gametes with 2*x* = 36 chromosomes (2*n*); dyad formation yields gametes with 3*x* = 54 chromosomes; and monad formation yields gametes with 6*x* = 108 chromosomes (Figure 4a–e). Among these, the *x* = 18 and 1.5*x* = 27 classes are expected to be the most frequent, as they arise from hexads and tetrads—the most common tetrad types observed (Figure 2m and Figure 4e). This theoretical expectation is consistent with the predominance of normal-sized pollen in our measurements (95% in the triploid). Notably, the 1.5*x* = 27 class represents aneuploid gametes, which are genetically unbalanced and likely non-functional, explaining why triploid rubber trees are rarely successful as male parents despite producing abundant morphologically normal pollen. In tetraploid rubber trees (2*n* = 4*x* = 72, where *x* = 18), the observed tetrad abnormalities provide a mechanistic explanation for the origin of different pollen size classes. Uneven tetrads (Figure 5h) are the most common abnormal type, producing four large microspores with 2*x* = 36 chromosomes (*n*) along with one or more microcytes containing fewer chromosomes. These large microspores develop into normal-sized pollen grains, which constitute the vast majority (95.23%) of the pollen population. The accompanying microcytes give rise to small pollen grains (4.75%), which are likely aneuploid and non-functional. Uneven dyads (Figure 5f) produce two large spores with 4*x* = 72 chromosomes (2*n*), which develop into large pollen grains, and two microcytes that develop into small pollen. Triads (Figure 5g) may produce three spores with variable chromosome numbers, potentially yielding a mixture of normal and large pollen grains. The extreme rarity of large pollen grains (0.025%) is consistent with the low frequency of dyad and triad formation in our observations. Thus, in tetraploid rubber trees, the predominance of normal-sized pollen reflects the high frequency of tetrad-type divisions that produce reduced (*n* = 2*x* = 36) gametes, while unreduced (2*n* = 4*x* = 72) pollen is rarely produced and largely non-functional due to abnormal morphology. Although pollen germination assays could not be performed due to sample limitations, we used acetic acid–magenta staining as an alternative indicator of cytoplasmic integrity [48]. Morphologically normal pollen grains of all size classes were uniformly stained, whereas small, shriveled, or malformed grains remained unstained, suggesting that unreduced (2*n* and 4*n*) pollen grains possess intact membranes and potential viability. However, membrane integrity is only one prerequisite for successful fertilization. The extreme rarity of these pollen classes (2.21% large and 2.59% super-large in triploids; 0.025% large in tetraploids), combined with their predominantly abnormal morphology, suggests that even if they possess intact membranes, they are unlikely to contribute substantially to functional male gamete pools in rubber tree polyploids.

An unexpected finding is that both triploids and tetraploids produced approximately 95% normal-sized pollen grains, with a diameter similar to that of normal diploid pollen (Figure 6a,d). This observation is counterintuitive because, theoretically, triploids should predominantly produce aneuploid and 2*n* gametes due to irregular chromosome segregation, whereas tetraploids are expected to generate a high frequency of 2*n* pollen as a result of balanced bivalent formation and reductional division. However, the present results suggest that in rubber tree polyploids, the majority of microspores undergo a seemingly normal reductional meiosis, yielding haploid pollen grains comparable in size to those of diploids. Several explanations may account for this. First, the rubber tree is a paleotetraploid that has undergone diploidization. It is possible that even in artificially induced triploids and tetraploids, the genome retains a tendency toward balanced chromosome segregation, favoring the production of euploid gametes with the basic chromosome number (*x* = 18). This interpretation is consistent with the observation of hexads at the tetrad stage in the triploid (Figure 4e), where each of the six microspores would theoretically receive *x* = 18 chromosomes if segregation were balanced. However, we emphasize that this interpretation is based on the morphological observation of hexad formation and does not constitute direct evidence for balanced chromosome segregation. We acknowldege that direct evidence for preferential bivalent pairing in these polyploids, such as chromosome configuration analysis at diakinesis or metaphase I, could not be obtained in this study due to technical limitations. The hexad formation observed is presented as a morphological observation consistent with the possibility of balanced segregation, not as proof thereof. Nevertheless, the hexad formation observed suggests that a fraction of microspores may receive a balanced chromosome complement and subsequently mature into haploid pollen. Second, abnormal meiotic products such as monads, dyads, triads, polyads, and microcytes may largely fail to develop into mature, functional pollen grains. Many of the larger pollen grains (putative 2*n* or 4*n*) observed in this study exhibited wrinkled, shriveled, or irregular shapes (Figure 4i,j and Figure 5m,n), suggesting they are nonviable. Consequently, only the normal-sized haploid pollen grains survive to maturity, leading to their overwhelming predominance. Third, in the tetraploid, small pollen grains derived from microcytes are likely aneuploid or lack a complete genome and are probably non-functional, whereas large pollen grains are extremely rare (0.025%). Therefore, despite the high frequency of abnormal tetrads in tetraploids (24.51%), the resulting pollen population is dominated by haploid pollen grains, which are similar in size to those of diploids. Collectively, our findings demonstrate that in rubber tree polyploids, the production of functional male gametes is largely restricted to haploid pollen, and that unreduced pollen, though morphologically identifiable, is either inviable or produced in negligible quantities. This explains why triploid and tetraploid rubber trees are rarely used effectively as male parents in crossing programs, although the underlying reasons may be more complex, and highlights the necessity of assessing both ploidy and viability when evaluating polyploid germplasm for breeding. Further studies on pollen germination and in vivo fertilization will be necessary to confirm the actual fertility of these different pollen classes.

We acknowledge two main technical limitations in this study. First, although acetic acid–magenta staining provided an indication of cytoplasmic integrity, we were unable to perform pollen germination assays to directly assess pollen viability due to the limited number of genotypes available and the loss of samples. Second, detailed chromosome configuration analysis at diakinesis and metaphase I could not be performed, as chromosomes at these stages were extremely condensed and densely packed in our fixed samples, preventing unambiguous identification of individual chromosome associations (univalents, bivalents, trivalents, or quadrivalents). This technical limitation is consistent with previous observations in *Hevea* cytogenetics [16,27,28,29,30,31], where the small size and large number of chromosomes have long posed challenges for detailed meiotic analysis. Future studies employing improved cytological techniques, such as synaptonemal complex spreading or FISH-based approaches, are needed to resolve chromosome pairing configurations in these polyploid cytotypes. Nevertheless, we believe these limitations do not detract from the main contributions of this study, which provides the first systematic description of triploid microsporogenesis and the first comprehensive quantitative analysis of pollen size variation in triploid and tetraploid rubber tree polyploids.

## 4. Materials and Methods

### 4.1. Plant Materials

The materials consisted of triploid and tetraploid rubber tree plants. The triploid is a progeny of Reken 525 (diploid) × Reyan 73397 (diploid), identified in our previous study [31]; it was obtained by artificial pollination in 2009 and planted at the National Rubber Tree Breeding Center base in 2010. The tetraploids comprised 14 plants discovered in an experimental field of the Chinese Academy of Tropical Agricultural Sciences; all 14 are derived from the same clone or are full siblings. The diploid clone Reyan73397 serves as one of potential parents, while the other parent remains unknown [49].

Ploidy levels were confirmed by flow cytometry in previous studies [31,49]. To further verify the exact chromosome numbers, mitotic chromosome counting was performed using unexpanded bronze-colored leaves following standard procedures. Leaves were pretreated with paradichlorobenzene-saturated solution for 4–6 h at room temperature, fixed in Carnoy’s fixative (ethanol:acetic acid = 3:1) for 24 h, hydrolyzed in 1 M HCl for 10–20 min, and stained with carbol-fuchsin solution. Cells with clear chromosome images were observed under an Olympus (Tokyo, Japan) BX53 microscope and photographed with a VP700c digital camera (Sony, Tokyo, Japan). At least 10 well-spread metaphase cells were counted for each plant.

In the springs of 2019 and 2020, the triploid plant and two tetraploid plants bloomed, and their inflorescences were collected. Inflorescences of the triploid were collected on 3 March and 7 March 2019, and again on 24 March 2020. Inflorescences of the tetraploids were collected on 8 March 2019 and on 25 March 2020. Whole inflorescences were fixed in Carnoy’s fixative (ethanol:acetic acid = 7:3, *v*/*v*) for 24 h at 4 °C and then transferred to 70% ethanol at 4 °C for long-term storage until cytological observation.

### 4.2. Observation of Microsporogenesis

Microsporogenesis was observed using temporary slides stained with acetic acid–magenta solution. Briefly, a male flower bud from a fixed inflorescence was placed on a glass slide, crushed with tweezers, and debris was removed. The sample was stained with a drop of acetic acid–magenta solution, covered with a coverslip, and examined under a Leica DMBL optical microscope (Leica, Wetzlar, Germany) to record meiotic stages. Microscopic images were captured with a Leica DFC550 camera (Leica, Wetzlar, Germany), and scale bars were added using ImageJ 1.52a software.

### 4.3. Observation and Measurement of Pollen

Mature but unopened male flower buds were randomly selected, and temporary slides were prepared as described above. All pollen grains on each slide were observed and photographed using a Leica DFC550 camera attached to a Leica DMBL microscope with a 10× objective lens. For each ploidy level, at least 10 buds were examined, and more than 10,000 pollen grains were counted. Pollen grains of different sizes and shapes were photographed using a 100× objective lens. Pollen grain diameter was measured using ImageJ 1.52a software. Because some pollen grains were not perfectly circular, the longest and shortest diameters of each grain were measured, and the diameter was calculated as the average of the two measurements. During the observation, pollen staining status was also recorded. Pollen grains with uniformly stained cytoplasm were considered as potentially viable, while those with unstained or shriveled appearance were considered non-viable.

Pollen grains were preliminarily classified into size classes based on visual inspection under the microscope [50], using the majority of normal-sized pollen grains in each field of view as an internal reference. Pollen grains that appeared conspicuously larger than normal pollen were tentatively designated as ‘large’, and those substantially larger than the large class were designated as ‘super-large’. Pollen grains visibly smaller than normal pollen were designated as ‘small’. Following this preliminary visual classification, the diameters of representative pollen grains from each class were measured using ImageJ software. The final classification of each pollen grain was confirmed by its measured diameter relative to the mean diameter of normal pollen from the same sample.

### 4.4. Data Statistics and Analysis

Data on abnormal pollen grains and pollen diameters were recorded in Microsoft Excel. Bar charts and scatter plots were generated using the ggplot2 package (v4.0.3) in R. Density distributions were plotted using the hist function in the graphics package (v4.4.2).

## 5. Conclusions

This study systematically characterized the morphological sequence of microsporogenesis and pollen morphology in triploid and tetraploid rubber trees. Both polyploids exhibited the complete morphological progression through meiotic stages, but with abundant abnormalities at the tetrad stage, including monads, dyads, triads, and uneven polyads. Tetraploids additionally showed chromosome misalignment, lagging chromosomes, and multipolar poles, with 24.52% abnormal tetrads. Despite these irregularities, approximately 95% of mature pollen grains in both polyploids were normal-sized and putatively haploid, comparable to those of diploids. Triploids produced 2.21% large (putative 2*n*) and 2.59% super-large (putative 4*n*) pollen, whereas tetraploids produced only 0.025% large pollen and 4.75% small pollen. Most large pollen grains exhibited wrinkled or shriveled morphologies, suggesting non-viability. The predominance of haploid pollen indicates that functional male gamete production is largely restricted to reduced gametes, explaining why triploid and tetraploid rubber trees are rarely successful as male parents in breeding. Given that functional pollen in polyploids is predominantly haploid, future efforts should prioritize ploidy verification via flow cytometry of individual pollen grains and in vivo germination assays to assess the true reproductive contribution of the rare unreduced pollen classes. Such data would be critical for designing interploidy crossing schemes, particularly using tetraploids as female parents to exploit 2*n* eggs.

## Figures and Tables

**Table 1:** The diameter of different types of pollen grains.

Ploidy	Types	Count	Min (μm)	Max (μm)	Mean ± SD (μm)
Triploid	Normal	229	30.71	43.30	37.19 ± 2.45
	Large	112	39.20	55.71	47.17 ± 3.82
	Super-large	149	56.55	75.06	64.16 ± 4.27
Tetraploid	Normal	201	31.66	46.65	38.39 ± 2.68
	Small	51	11.76	29.20	19.59 ± 4.21
	Large	3	54.17	56.71	55.30 ± 1.29

## Data Availability

Data are contained within the article.
